# Equality, Diversity and Inclusion characteristics measured or reported in randomised trials of intrapartum interventions: A Scoping Review

**DOI:** 10.12688/hrbopenres.14012.1

**Published:** 2024-12-24

**Authors:** Susan Hannon, Aoife Smith, John Gilmore, Valerie Smith

**Affiliations:** 1 School of Nursing and Midwifery, The University of Dublin Trinity College, Dublin, Leinster, D02, Ireland; 2 School of Agriculture and Food Science, University College Dublin School of Nursing Midwifery and Health Systems, Dublin, Leinster, D04, Ireland; 3 School of Nursing, Midwifery & Health Systems, University College Dublin, Dublin, Leinster, D04, Ireland

**Keywords:** Intrapartum, Labour, Childbirth, Equality, Diversity, Inclusion, Randomised trials

## Abstract

**Background:**

Equality, diversity and inclusion (EDI) has gained discursive momentum across multiple arenas, including in maternal health research. As a preliminary exploration for future discussion and development, we undertook a scoping review to identify the types, frequency, and extent of EDI characteristics that were measured and reported in randomised controlled trials (RCTs) of intrapartum interventions specifically.

**Methods:**

Joanna Briggs Institute methodological guidance for scoping reviews guided the conduct of the review. The population were women of any parity and risk category who were enrolled in intrapartum RCTs in any birth setting or geographical location. The concept was measured and reported EDI characteristics. CINAHL, MEDLINE, PsycINFO, EMBASE, and CENTRAL were searched from January 2019 to March 2024. Data were extracted using a pre-designed form. The findings were summarised and narratively reported supported by illustrative tables and graphs.

**Results:**

Two-hundred and forty-seven RCTs from 49 countries were included. Eleven EDI characteristics were measured or reported in at least one RCT, although frequency varied. Religion, for example, featured in three RCTs only, whereas Age featured in 222 RCTs. How the EDI characteristics featured also varied. Race/Ethnicity, for example, was described in 21 different ways in 25 RCTs. Similarly, Education was reported in 62 different ways across 96 RCTs. Ninety RCTs limited inclusion to nulliparous participants only, six RCTs required participants to have a minimum educational level, 127 RCTs had inclusion age cut-offs although 23 different variations of this were noted and 15 RCTs excluded participants on the grounds of disability.

**Conclusions:**

This scoping review highlights EDI characteristic measurement and reporting deficits in intrapartum RCTs. There is a critical need for improvements in designing, conducting, and reporting RCTs to incorporate EDI. By adopting more extensive EDI practices a greater understanding of healthcare treatments and innovations leading to enhanced maternal health equity could be achieved.

## Introduction

Equality, diversity and inclusion (EDI) has increasingly gained discursive attention across multiple arenas, for example, in education, employment, and in healthcare practice and research. EDI as an overarching concept exists in multiple phraseology formats in policy and across societal sectors, encompassing individual discrete components (see Table 1) although associated and interrelated, for which a communal consensus definition is lacking ^1^. Viewing EDI ontologically and axiologically may bring some clarity whereby the ethical and moral issues of social, structural and systemic inequalities and lack of representation of marginalised or underserved populations is considered ^2, 3^, and potentially addressed by meaningfully applied EDI practices ^4^.

**Table 1.** EDI component definitions ^29^.

Component	NIHR Definition	Applied to the Scoping Review
Equality	Ensuring that everyone is given equal access to resources and opportunities to utilise their skills and talents.	Equal opportunities and access for all eligible persons to participate in intrapartum RCTs
Diversity	Being reflective of the wider community. Having a diverse community, with people from a broad range of backgrounds represented in all areas and at all levels.	Representation of people from a range of backgrounds in intrapartum RCTs
Inclusion	An approach where groups or individuals with different backgrounds are welcomed, culturally and socially accepted, and treated equally. Engaging with each person as an individual. A sense of belonging that is respectful of people for who they are.	RCT environments are supportive towards participation by all those eligible, that is, strategies to support participation by those, for example, with low literacy or disability (unless prohibitive due to the treatment being studied)

In healthcare, socio-ecological factors, ethnicity, age, sex, and physical health factors are known to play a role in the experience of ill health, ability to access healthcare, treatment response, and short and long-term outcomes ^5– 10^. Pragmatically, this knowledge provides motivation for insisting on EDI in healthcare research. Randomised controlled trials (RCTs) are considered the gold standard for evaluating cause and effect relationships between treatments and health outcomes ^11^. The results of RCTs should have relevance to all populations that may potentially benefit from the treatments being studied. This requires participation and representation of individuals of diverse backgrounds to ensure that RCT findings are generalisable, that the treatments or interventions are suitable for the populations to which they will be applied, and that illness impact and progression and treatment effectiveness may be understood and contextualised by differences related to potential diversity ^12– 14^. Historically, however, populations who represented those most affected by a disease or health condition or for which interventions were most needed were not always recruited to RCTs thus limiting the translation of findings to real-world settings ^15^. Underrepresentation in RCTs has implications too for the ethical conduct of research, whereby the principles of beneficence, non-maleficence, justice and veracity may be undermined.

The enrolment and participation of fewer women in RCTs, as an illustrative example, renders sex-based differences in disease aetiology, epidemiology, pathogenesis, and treatment effects to remain hidden or undiscovered ^16– 18^. Women who are pregnant or lactating are also frequently excluded from medical RCT participation due to prohibitive regulatory barriers ^19, 20^. These exclusions have real-world implications whereby the impact of a treatment or intervention remains unknown for these populations with a consequential lack of access to health interventions that may be effectively experienced by the wider population. The COVID-19 pandemic provides a further, albeit contrasting example. In the early phases of the pandemic, it became known that racial and ethnic minority populations were being disproportionately affected by the virus. By being inclusive of all populations, vaccine trial researchers were able to demonstrate the efficacy of COVID-19 vaccine across ethnic and minority groups, thus offering a safe and effective intervention irrespective of racial or ethnic origin ^21^, although some trials were criticised for their lack of inclusivity of diverse ethnic and marginalised populations ^22^.

Health disparities feature prominently in maternity care. Recent data from the UK highlights considerable disparities in maternal health outcomes in women of diverse ethnicity, for example, the mortality rate from pregnancy to 6-weeks postpartum in Black, Asian, and women of mixed ethnicity was reported as being four, almost two, and three times that of White women, respectively ^23^. Black, Asian and minority ethnic (BAME) women are also more likely to experience a stillbirth ^24^, pre-eclampsia ^25^, severe maternal morbidity ^26^, and have an increased risk for preterm birth ^27^. Despite this awareness, Black women remain underrepresented in maternal health research, and women of other ethnic minorities, such as women who are Asian, American Indian or Alaskan native, Hispanic, Aboriginal/Torres Strait Islander, or Native Hawaiian and other Pacific Islanders are even less represented ^28^.

Further confounding under-representation is the extent to which results based on participants differ from those of all eligible participants, and the implications of this for clinical practice. This was particularly highlighted following publication of the ARRIVE trial, a RCT that evaluated elective induction of labour (IOL) during gestational week 39 compared to expectant management at ≥40+5 weeks in low-risk nulliparous women with a singleton fetus in the vertex position ^30^. The RCT, which involved 41 hospitals in the United States (US), demonstrated a 20% non-significant reduction in the primary outcome (composite of severe neonatal complications or mortality) and a significant reduction in caesarean birth in the IOL group (18.6% versus 22.2% respectively, p<0.01). A subsequent statement published by the Society for Maternal-Fetal Medicine (SMFM) recommended that “ *It is reasonable to offer elective induction of labor to low-risk, nulliparous women at or beyond 39 weeks and 0 days of gestation”* and that “ *women can be reassured that both elective IOL and expectant management are reasonable options at 39 weeks of gestation*” ^31^. Concerns related to ARRIVE’s applicability and transferability to other settings, however, were cited across several subsequent publications ^32– 34^. These concerns included an overall caesarean birth rate in ARRIVE that was considerably lower than the US national average, a higher proportion of African-American participants than that of the wider US birthing population (23% versus 15% respectively), proportionately fewer women ≥35 years of age than usual; 4% in ARRIVE versus 18% in US, and 60% and 10% in Europe in the categories of 30–39 and ≥40 years, respectively ^34, 35^, and a higher proportion of women with a pre-pregnancy Body Mass Index >30; 53% in ARRIVE versus 14% in European maternity populations ^34^.

Guidance for trialist on inclusive practices to help address and overcome issues associated with selection bias and population under-representation in RCTs are available ^29, 36^, and will likely further develop as focused momentum continues. The INCLUDE Ethnicity Framework, for example, *‘aims to help trial teams think specifically about which ethnic groups should be included in their trial’*, while acknowledging that ethnicity is complex, and may depend on aspects such as the ‘ *geographic location of the trial, and the disease or condition targeted’* ^37^. The Framework includes four key questions for trialists to consider when thinking about who participants should be, and how to facilitate their involvement ^36^. While such guidance has broad applicability, there is a need also to consider EDI characteristics beyond and in addition to ethnicity, and to consider EDI characteristics specifically in the context of RCTs involving discrete populations such as those who are pregnant or postpartum. For this reason, to add to the discourse on EDI in RCT research and as a preliminary exploration for potential future developments, we conducted a scoping review to identify the types, frequency, and extent of EDI characteristics that were measured and reported in published RCTs of intrapartum interventions. For purposes of this review, we considered EDI under the definitions offered by the UK’s National Institute for Health and Care Research (NIHR) EDI Strategy 2022–2027 ^29^, interrelated at the component level and in the context of participation in intrapartum RCTs ( Table 1). Twelve EDI characteristics of interest were identified for this review by drawing on the Protected Characteristic in the UK Equality Act 2010 ^38^, the NIHR EDI Strategy ^29^, and the Diversity and Inclusion Survey Question Guidance ^39^ ( Table 2).

**Table 2.** EDI characteristics of interest.

Characteristic	Source(s)
Race/Ethnicity	1, 2, 3
Age	1, 2, 3
Relationship status (marriage/civil partnership, single mothers)	1, 2
Language	2
Education level (includes literacy)	2, 3
Religion	1, 2, 3
Socio-economic status	2, 3
Rural/Urban population	2
Sexual Orientation	1, 2, 3
Gender	1, 3
Co-morbidities	3
Disability ( *physical, ID,* n *eurodiversity*)	1, 2, 3

1=Protected Characteristic in the UK Equality Act 2010 2=NIHR Equality, Diversity and Inclusion Toolkit 3=Daisy Question Guidance

## Review objectives

The objectives of the scoping review were to:

1. Identify and describe the types (which ones) of EDI characteristics being measured and reported in intrapartum RCTs.
2. Identify and describe the frequency (how often) that EDI characteristics are being measured and reported in intrapartum RCTs.
3. Identify and describe the extent of measurement and reporting, that is, at what stage(s) of intrapartum RCTs are EDI characteristics embedded, for example, as part of the inclusion or exclusion criteria, as a baseline characteristic, or as an outcome in analysis.

## Methods

The protocol for this scoping review was registered prospectively in Open Science Framework (OSF) ( https://osf.io/7qmd2/). The Joanna Briggs Institute (JBI) methodological guidance for scoping reviews was used to guide the conduct of the review ^40^. For reporting the review, we adhered to the Preferred Reporting Items for Systematic Reviews and Meta-Analyses extension for Scoping Reviews (PRISMA-ScR) ^41^ (Extended File 1: https://osf.io/7qmd2/). 

### Inclusion criteria

The Population, Concept, Context (PCC) framework for scoping reviews ^40^ was used to frame the review’s inclusion criteria.

Population: Women of any parity and risk category enrolled in RCTs of any design (parallel, cluster, cross-over) during intrapartum care. We chose to limit the review to the intrapartum period primarily to provide clinical homogeneity in the maternity stage in which the RCTs were occurring, while also maintaining manageable scope in conducting the review. The intrapartum period, for purposes of the review, was defined using the National Institute for Health and Care Excellence (NICE) definition for established first stage of labour through to the end of the third stage of labour, that is regular uterine contractions and progressive dilatation of the cervix from 4cms dilated until birth of the baby and expulsion of the placenta and membranes ^42^. RCTs that enrolled women and commenced the intervention before established labour, for example, antenatally or in early labour (women with a cervical dilatation of 3cm or less), even if the intervention continued through to established labour, were excluded. Similarly, if it was unclear whether women were in established labour at the point of enrolment, these RCTs were excluded. RCTs that enrolled women in late second stage of labour with an intervention that was applied before birth of the placenta and membranes even if the outcomes were measured postpartum or were specific to the neonate, were included. 

Concept: EDI characteristics ( Table 2) as measured and reported in the included RCTs.

Context: The context was completed and reported (published) RCTs of any design that evaluated an intrapartum intervention in any birth environment (i.e., hospital, midwifery-led units, home), in any geographical location. Trials of designs other than randomised, for example, quasi-experimental or clinical non-randomised trials of interventions, were excluded.

### Search and selection strategy

To retrieve relevant RCTs the electronic databases of CINAHL (EBSCO), MEDLINE (OVID), PsycINFO (OVID), EMBASE (OVID), and Cochrane Central Register of Controlled Trials (CENTRAL) were searched from January 2019 to March 2024. We limited our search to the past five years to maintain contemporaneous scope in the conduct of the review. As part of our search strategy, we adopted the Cochrane Highly Sensitive Search Strategy ^43^ for identifying RCTs and adjusted this to conform to each database’s search format. The search terms and combinations of these are presented in Extended File 2 ( https://osf.io/7qmd2/). Retrieved citations were downloaded initially to EndNote (Version 20.5, Clarivate Analytics) where duplicate records were removed. As we were interested in completed and reported (published) RCTs, grey literature searching was limited to a search of trial registries based only on the trial registry number provided in the included RCT reports. Language restrictions were not applied to the search strategy; however, due to a lack of translation services, only RCTs published in English were included. Searching without language restrictions, however, enabled us to evaluate the potential for language bias as a possible limitation in the review.

Retrieved records, following de-duplication in Endnote, were uploaded to Covidence for screening. To ensure inter-rater reliability between reviewers, two reviewers initially screened 10% of retrieved records (by title and abstract) against the review’s inclusion and exclusion criteria, using the AMSTAR-2 recommendation of at least 80% agreement as an acceptable congruency cut-off ^44^. Agreement was >90%, and one reviewer thus screened the remaining title and abstracts, excluding those that were clearly ineligible, and forwarding the remainder for full-text screening. Two reviewers independently screened all records at full text level. Any uncertainties or disagreements were resolved through discussion and consensus.

### Quality appraisal

As the aim of the scoping review was to identify and describe the types, frequency and extend of EDI characteristics, individual RCT quality was not of particular concern. In this regard, a methodological quality appraisal of the included RCTs was not performed which is in line with JBI guidance for the conduct of scoping reviews ^40^. 

### Data extraction

Data were extracted using a purposively designed data extraction form (DEF) constructed in Microsoft Excel (Extended File 3: https://osf.io/7qmd2/). The DEF was piloted by pairs of reviewers independently on 20 of the included records to assess the form’s suitability, and to cross-check accuracy and congruency in extracting the data. As data extraction congruency was almost 100%, discussions following the pilot centred on clarifications rather than revisions. The remaining records, following the pilot, were divided amongst the same three reviewers, and each reviewer extracted the data from their allocated records. Prior to and in preparation for data charting, one reviewer assessed all extracted data in the DEF for consistency in how the extracted data were recorded, and standardised any data as required; for example, if one extractor had used the full term for a clinical condition from one RCT (e.g., umbilical cord clamping) and a second had recorded an abbreviated term for the same condition from another RCT (e.g., UCC), this was standardised to the same format for both RCTs.

Information extracted to the DEF included the RCT reference details, registration details, dates RCT was conducted, country of origin, RCT design, aim, setting, clinical condition, ethical approval information, total participants included in the analysis, and whether each of the EDI characteristics ( Table 2) were measured or reported as part of sample characteristics, inclusion/exclusion criteria, or data analyses. We also extracted information related to gravida/parity representing pregnancy and maternity in the UK Equality Act 2010 ^38^.

### Data charting and presentation of the findings

As recommended for scoping reviews, rather than providing a statistical or formal thematic synthesis of data, summary findings narratively supported by illustrative charting using tables and graphs, were presented. One reviewer undertook the charting processes, and a second reviewer aligned this charting with associated narrative summaries to address the review objectives.

## Results

### Search and selection results

**Figure 1.**
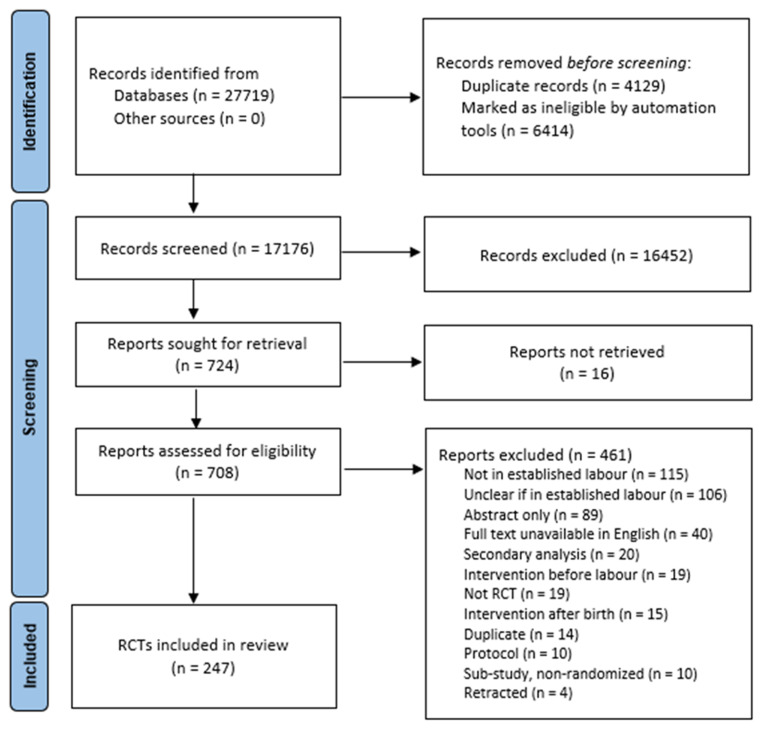
Search and Selection PRISMA Flow Diagram ^45^.

The search strategy retrieved 27,719 records, of which 10,543 were duplicates or marked ineligible in Covidence by the RCT automated filter and were removed. Title and abstract screening of 17,176 records occurred of which 16,452 were excluded as ineligible. Of the remaining 724 records, we could not obtain the full text for 16 of these. The full texts of 708 records were thus screened, of which 461 were excluded with reasons documented ( Figure 1). This provided 247 eligible RCTs that were included in the review. Figure 1, publicly available for download and use via the Equator Network ( https://www.equator-network.org,/reporting-guidelines/prisma/) illustrates the search and screening process.

### Characteristics of included trials

**Figure 2.**
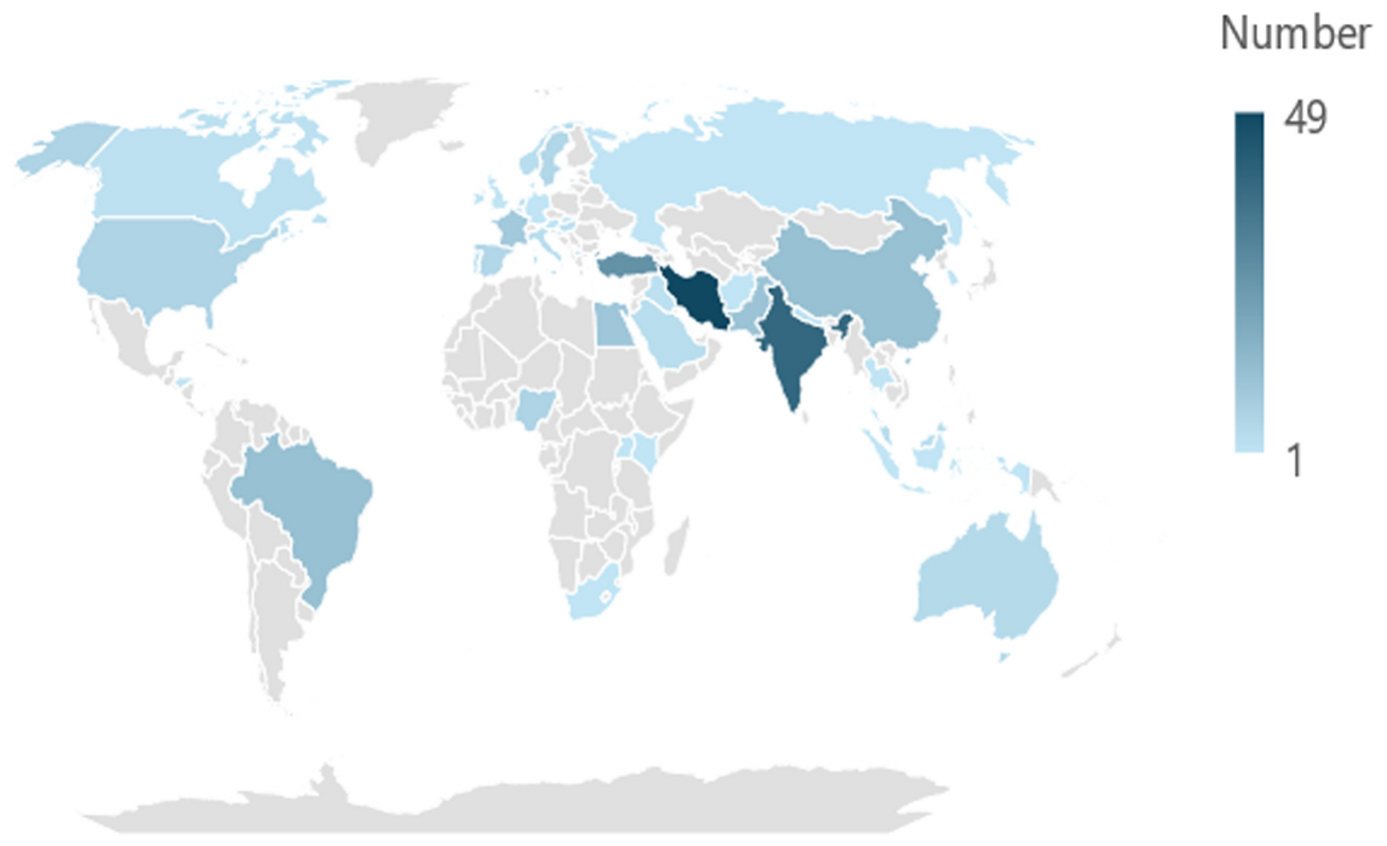
Country of origin of included trials.

The country of origins of the included RCTs represented a global spread ( Figure 2). The largest number of RCTs originated in Iran (n=49), followed by India (n=39), Turkey (n=26), Brazil (n=12), China (n=12) and Pakistan (n=11). A further nineteen countries each contributed one RCT to the dataset. Six countries each contributed two RCTs, five countries three RCTs, Australia contributed four RCTs, Spain and Sweden contributed five RCTs each, France nine RCTs, Egypt 10 RCTs, and for seven RCTs, the country of origin was unknown.

**Figure 3.**
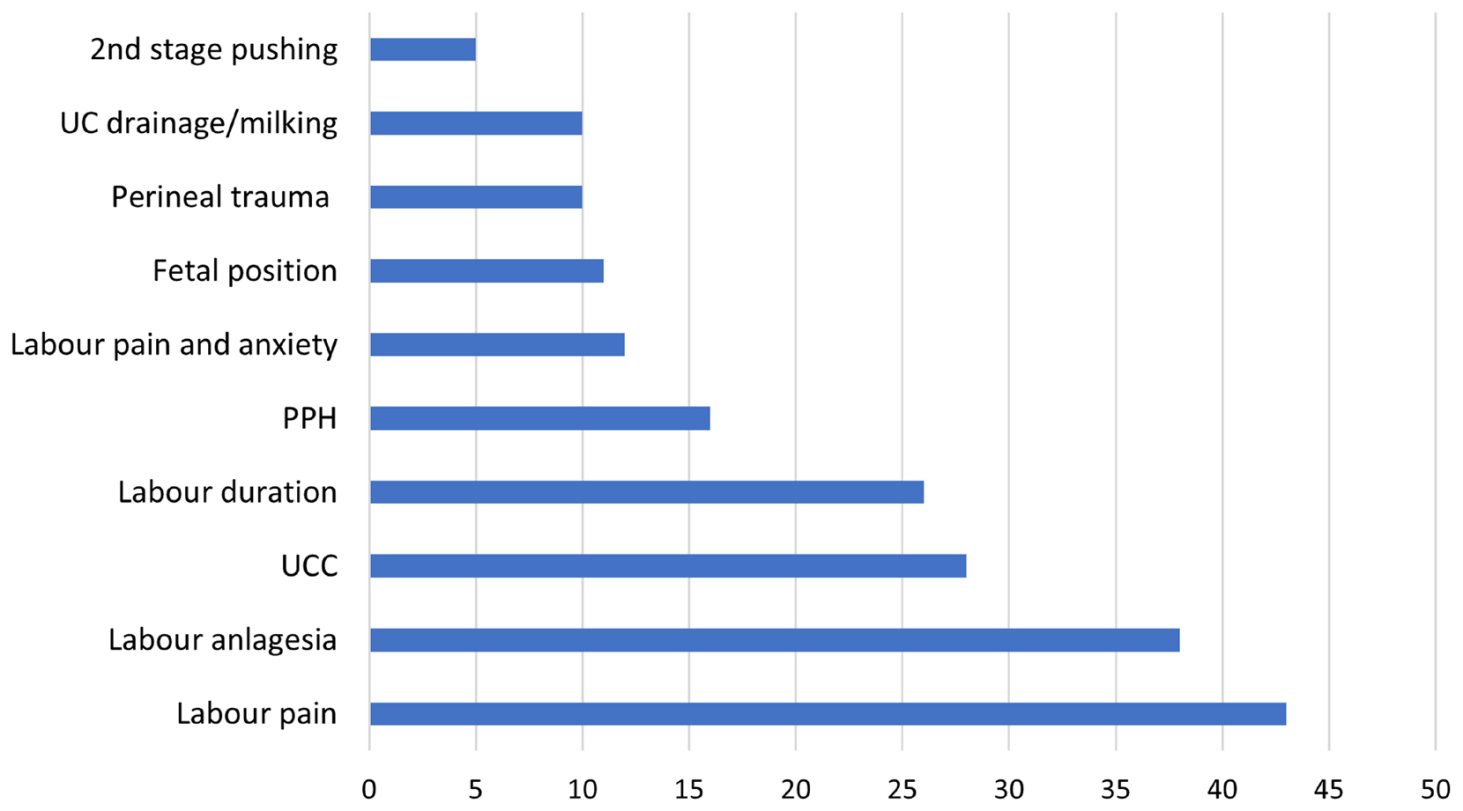
Trial clinical conditions.

RCT design was described in all 247 included RCTs, albeit in 63 different ways. Most were described simply as a ‘randomised controlled trial’ (n=113, 46%), followed by ‘double-blind randomised controlled trial’ (n=27, 11%). Variations in how the RCT designs were described in the remaining RCTs, although subtle, was extensive. Extended File 4, Table S4.1 ( https://osf.io/7qmd2/) illustrates this variation across the 247 RCTs. The RCT start dates spanned 1994 to 2023, with duration periods ranging from one month to seven years. Forty-two RCTs did not report the trial conduct dates. The total number of participants in the 247 included RCTs was 57,953 with a range of 10 to 4,913, mode of 100 and mean of 260. Forty-seven broad intrapartum topics or conditions of interest were identified in the 247 RCTs. Labour pain (n=43, 17%) and labour analgesia (n=38, 15%) featured most frequently, followed by umbilical cord clamping (n=28, 11%), and labour duration (n=21, 9%). Figure 3 illustrates the top 10 most frequently occurring topics/conditions and the numbers of RCTs that reported on these.

### Objectives 1 and 2: Types and frequencies of EDI characteristics

**Figure 4.**
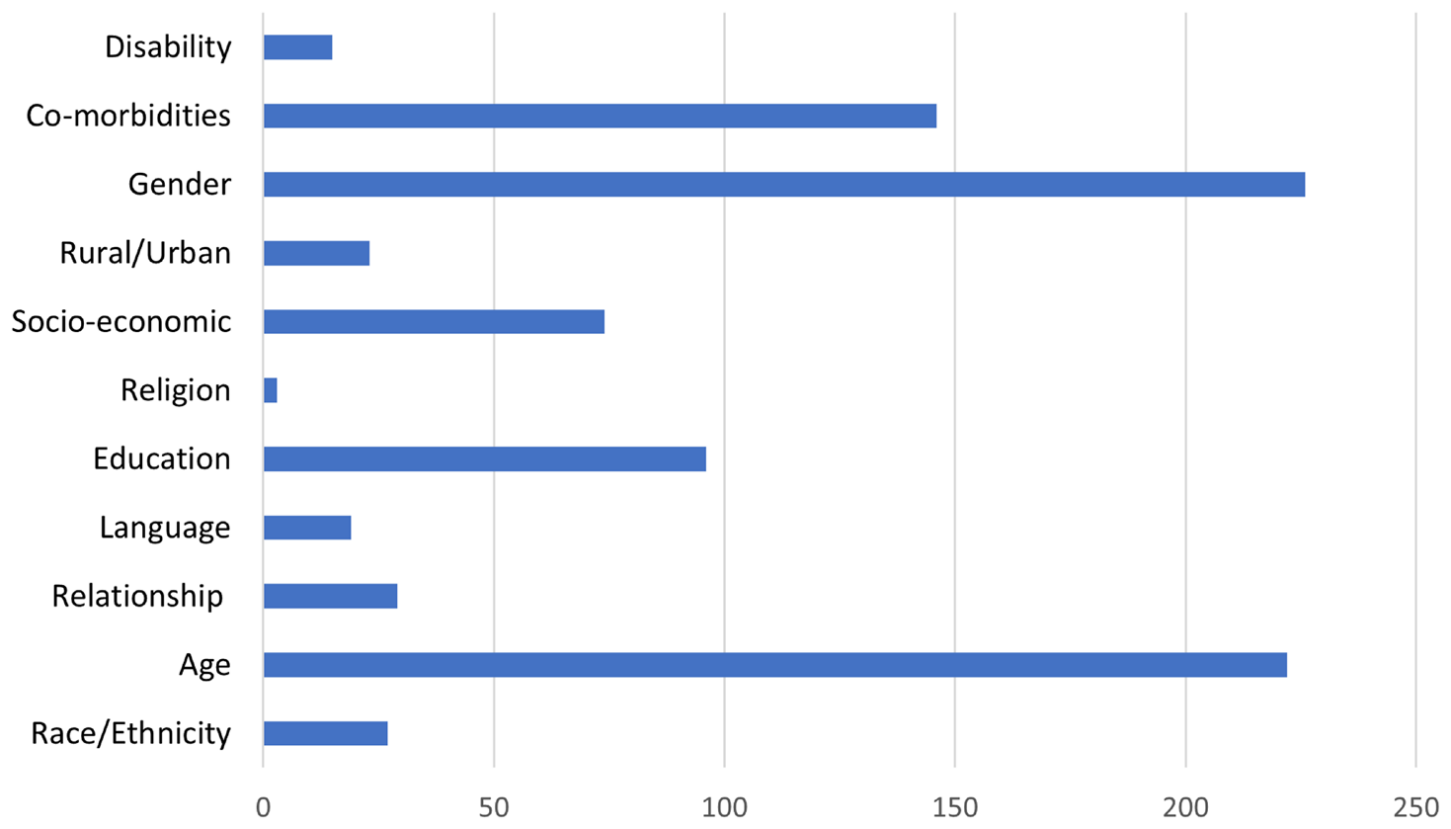
Numbers of trials that measured or reported each EDI characteristic.

Eleven of the 12 EDI characteristics were measured or reported in at least one RCT. The characteristic of Sexual Orientation did not feature in any RCT. The frequency of EDI characteristic measurement and reporting was varied. Religion, for example, featured in three RCTs only, whereas Age was measured and reported in 222 RCTs. Figure 4 illustrates the number of RCTs that measured or reported the 11 EDI characteristic that appeared in at least one RCT.

**Figure 5.**
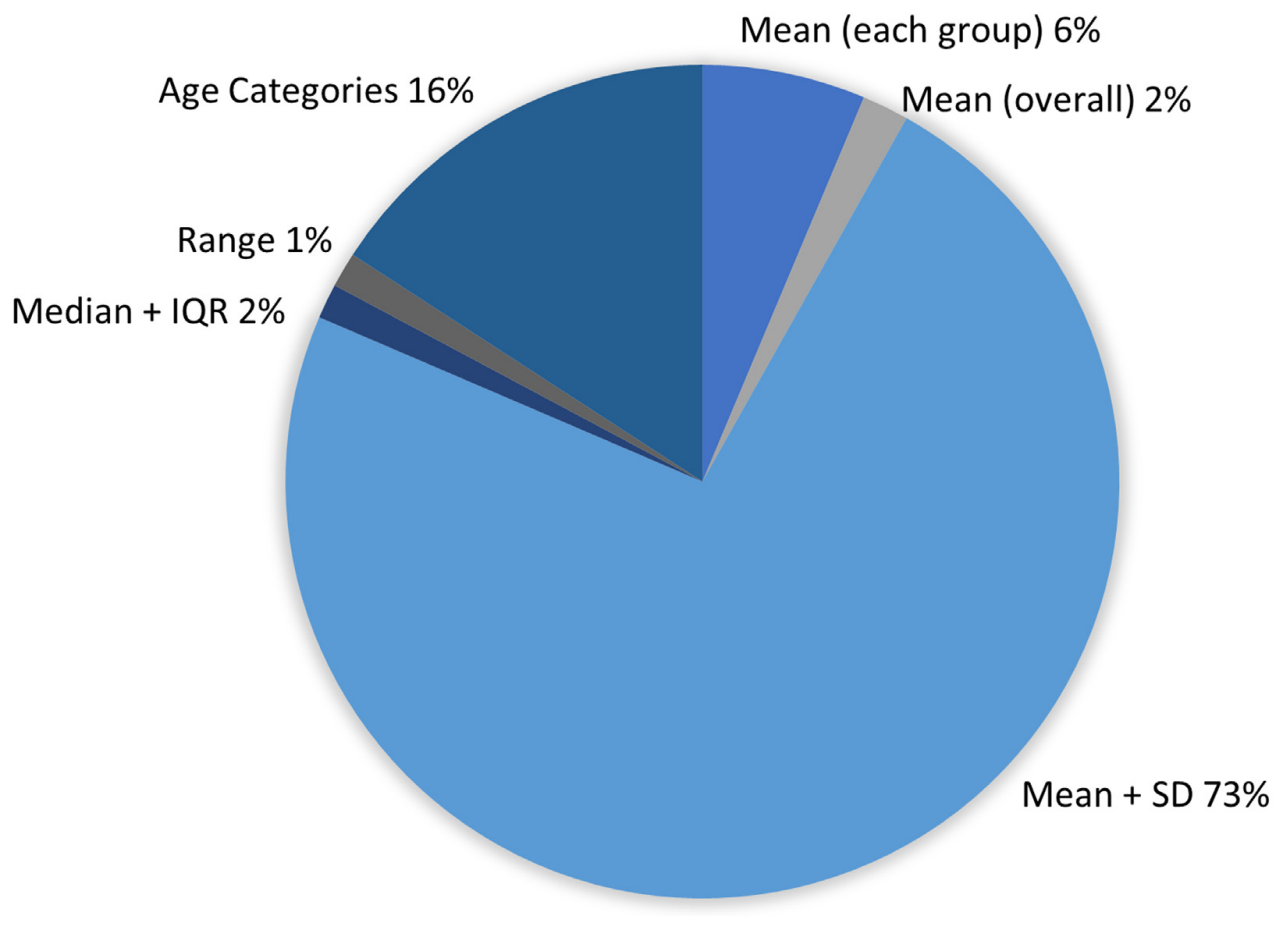
Proportion of RCTs reporting each Age broad descriptor.

Descriptions of the EDI characteristics also varied extensively. For example, Race/Ethnicity was described in 21 different ways ( Table 3). For maternal Age, although five main reporting descriptors were identified ( Figure 5), within the Age categories descriptor, 31 different categorisations were noted (Extended File 4, Table S4.2, https://osf.io/7qmd2/). Similarly, for Education, which featured in 96 RCTs, this characteristic was measured and reported in 62 different ways (Extended File 4, Table S4.3, https://osf.io/7qmd2/). For descriptions of the remaining EDI characteristics, see Extended File 4, Tables S4.4 to S4.6 ( https://osf.io/7qmd2/).

**Table 3.** Race/Ethnicity Descriptors.

**Race/Ethnicity Descriptors**	Description
	Tribe (Yoruba, Igbo, Hausa, Other)
	Caucasian only
	Caucasian, Other
	Caucasian, Black, Asian, Brown-skinned
	Dutch, non-Dutch
	Most women self-declared as White
	European, Southeast Asian, South Asian, Other
	Region of birth: France, Africa, Asia, Other
	Nordic, European, African, Middle Eastern, South American, Asian
	Spanish, non-Spanish
	Colour - White, Black, Yellow, Brown
	Malay, Chinese, Indian, Others
	Born in France, Other
	Black, White, or none of the above
	White, not White
	Mother’s birthplace: Spain, Latin America, Eastern Europe, Western Europe, Africa, Oceania, elsewhere
	White, Southeast Asian, South Asian, Other
	Caucasian, not white
	Hispanic or Latino, American Indian or Alaskan Native, Asian, Black or African American, White, Other
	Black or African American, White, Hispanic or Latina
	White, African, Other

### Objective 3: Stage(s) of RCTs that EDI characteristics were embedded

Three stages of a RCT for EDI measurement and reporting were explored in this scoping review. These were as a baseline sample characteristic, as an inclusion or exclusion criterion, or as part of analyses. Table 4 illustrates the findings for the 11 EDI characteristics that were embedded in at least one stage of at least one included RCT.

**Table 4.** Stage of trial that EDI characteristic (n=11) was measured or reported.

EDI characteristic	Sample characteristic n/247 (%)	Inclusion or Exclusion criterion n/247 (%)	Data analysis * n/n * (%)
Race/Ethnicity	23 (9.3)	4 (1.6)	20/23 (86.9)
Age	221 (89.5)	127 (51.4)	209/221 (94.6)
Relationship status	29 (11.7)	2 (0.8)	25/29 (86.2)
Language	1 (0.4)	18 (4.2)	1/1 (100)
Education Level	91 (36.8)	6 (2.4)	82/91 (90.1)
Religion	2 (0.8)	1 (0.4)	2/2 (100)
Socio-Economic Status	74 (29.9)	2 (0.8)	70/74 (94.6)
Rural/Urban Population	23 (9.3)	0	22/23 (95/6)
Gender	153 (61.9)	196 (79.4)	5 *
Co-morbidities	51 (20.6)	114 (46.2)	39/51 (76.5)
Disability	0	15 (6.1)	0

* All data analyses were based on baseline sample characteristics (groups compared at baseline), hence the denominator used in the Data analysis column is the sample characteristic numerator, other than for gender where comparisons by group for infant gender were presented.

**Figure 6.**
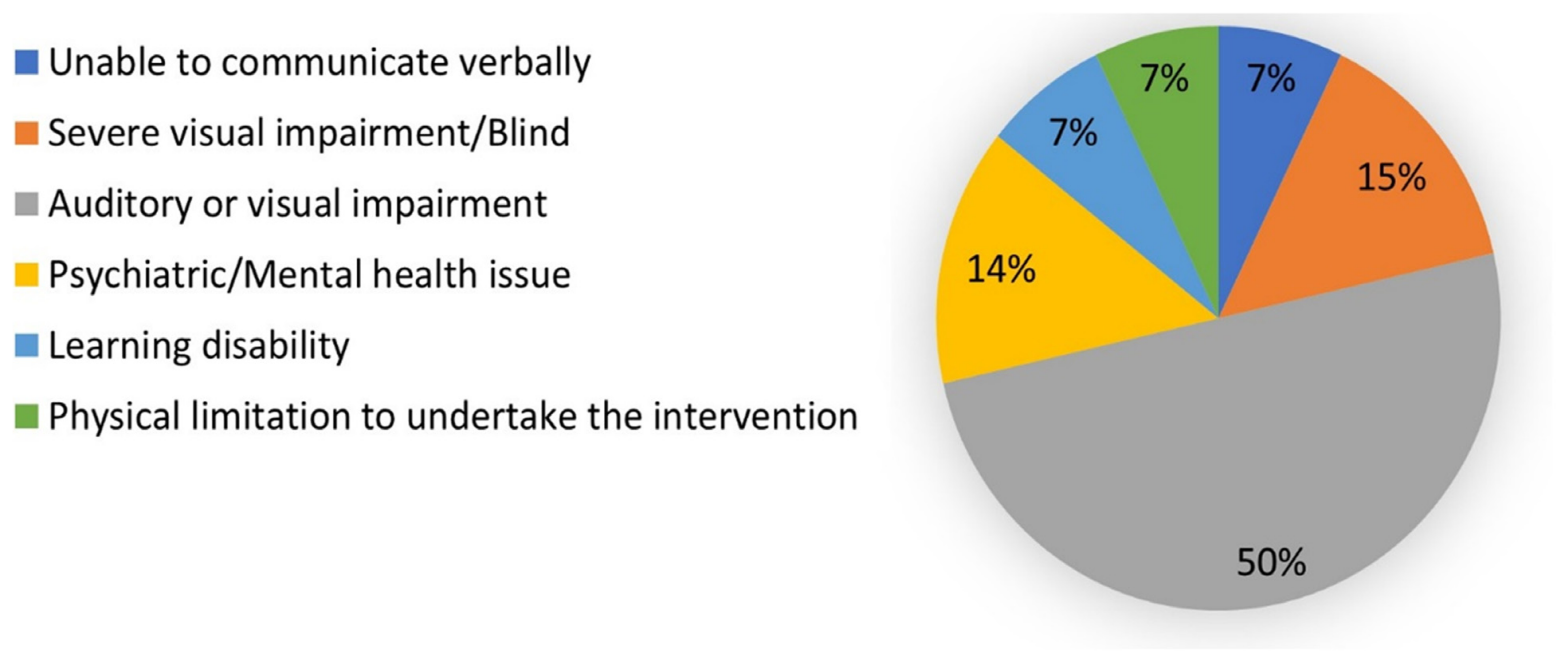
Disability (exclusion criteria).

With regards to inclusion/exclusion criteria, one RCT only had Religion (Muslim only) reported as an inclusion criterion. Six RCTs had a required minimum educational level (being literate, having minimal diploma literacy, minimum reading or writing literacy, educated to primary level or above, and ability to understand the intervention or the study information). Gender featured highly in sample inclusion/exclusion criteria, most frequently denoted as ‘women’ (n=193), ‘female women’ (n=1) and ‘pregnant females’ (n=2). Non-gender descriptions included ‘patients’, ‘parturient’, ‘participants’ or by parity or gravida, that is ‘Nulliparous’, ‘Primiparous’ or ‘Primigravida’. In six RCTs, the Gender of the infant was also described in the sample characteristics as ‘desired gender of the baby’ (n=1), ‘gender of the infant’ (n=4) and ‘male infant gender’ (n=1). Of these six trials, five compared Gender of the infant for each group at baseline. Fifteen RCTs excluded participants on the grounds of Disability ( Figure 6). For the EDI characteristics of Age, 127 reported cut-offs for age as an inclusion/exclusion criterion, although 23 different variations of this were identified ( Table 5).

**Table 5.** Variation in Age inclusion/exclusion criterion.

No. of trials	Description (years)	No. of trials	Description (years)
34	18–35	2	18–34
27	18	2	19–45
22	≥18	2	Any Age
7	18–40	1	19
4	18–45	1	19+
4	20–40	1	15–45
3	19–35	1	18–42
3	20–30	1	18–47
3	20–35	1	18–49
2	<35	1	18–50
2	>16	1	20–27
2	16–40		

Similarly, of the 18 trials that had Language as an inclusion or exclusion criterion, 12 variations of this were noted ( Table 6).

**Table 6.** Inclusion/exclusion Language variations.

No. of trials	Description
3	Included if literate, proficient in, or able to communicate in the Turkish language
1	Included only if a Kurdish speaker
1	Included if can consent in English and is proficient in Swedish, English, Arabic, or Farsi
2	Must have English language
1	Must have French language
1	Must have English or Afrikaans
1	Must be Swedish speaking
1	Excluded if language barrier
3	Excluded if unable to communicate in the Dutch language
1	Excluded if unable to speak or understand French
1	Excluded if unable to communicate fluently in French
2	Excluded if poor communication skills in Norwegian or English

Body Mass Index/obesity, hypertension and diabetes were the Co-morbidities most frequently reported in sample characteristics across the included RCTS, with 39, ten and seven RCTs respectively reporting on one or more of these conditions. Almost half of the RCTs (46.2%) had co-morbidities as an inclusion/exclusion criterion. While these co-morbidities varied extensively, most related to high-risk pregnancy and pre-existing medical conditions, such as cardiovascular, respiratory, neurological, renal or liver conditions. In 14 RCTs, a history of substance abuse/drug addiction featured as an exclusion criterion, and in 23 RCTs women with a previous or current history of mental health issues or psychiatric disorders were excluded. Gravida or parity were also described in the inclusion criteria of 106 RCTs. Notably, nulliparous or primiparous featured considerably, that is nulliparous, primigravida or primiparous women only were included in 85% of the 106 that noted Gravida/Parity in their inclusion or exclusion criteria.

## Discussion

This is the first comprehensive exploration, known to the authors, of the types, frequency and extent of EDI characteristic measurement and reporting in intrapartum RCTs. Being cognisant of the importance of EDI in health research, we recognise also that participation in RCTs is complex and often context dependent. Nonetheless, we found considerable variation in measurement and reporting of our 12 pre-specified EDI characteristics in this scoping review.

The first notable variation was in frequency of the number of trials that measured or reported each characteristic which ranged from three (Religion) to 226 (Gender), revealing that some EDI characteristics are receiving greater attention than others. Sexual Orientation as an EDI characteristic was entirely absent, yet research has shown disparities in pregnancy and birth outcomes based on Sexual Orientation data. Bisexual and lesbian women, for example, are more likely to experience miscarriage (Odds Ratio (OR) 1.77, 95% Confidence Interval (CI) 1.34 to 2.35), stillbirth (OR 2.85, 95% CI 1.40 to 5.83) and preterm birth (OR 1.84, 95% CI 1.11 to 3.04) than heterosexual women ^46^. Given that some of the included RCTs were conducted in countries where homosexuality remains criminalised (e.g., Iran, Pakistan), non-measurement or reporting might be understandable in these RCTs, but not for those outside of such jurisdictions. Gender, alternatively, featured in almost 80% of the included RCTs albeit using the term women, with no assurances of sex and gender separation. Pezaro and colleagues argue that gender-inclusive language is a safety and communication-critical issue is maternity care and suggest that use of the term women is exclusive, suggesting alternatively that pregnant people, pregnant populations and other non-genderised terms should be used ^47^. Beyond terminology, collecting sexual orientation and gender identity (SOGI) data, is considered necessary for monitoring and addressing health disparities, yet several prohibitive barriers to this have been identified: these include lack of awareness of sexual orientation and gender diversity, lack of institutional support, infrastructure workflow, discomfort from the clinician perspective, discomfort from the participant perspective and lack of training ^48^. Poor integration of SOGI data across electronic health records has also been highlighted as a major barrier to EDI for sexual minorities in clinical research, as well as a risk for misgendering, inappropriate questioning or inappropriate medical intervention ^49^.

The review also found minimal reporting of Race/Ethnicity in sample characteristics (9% of 247 RCTs), emulating that found in other studies ^50, 51^, and an absence of outcome evaluation by Race/Ethnicity in sub-group analyses. This was surprising given that many EDI guidelines or toolkits place emphasise on this characteristic ( Table 2), and there is evidence for disparate health adversity across racial and ethnic groups ^23– 25^. Considering the recent momentum and focus on EDI in RCTs, however, it remains to observe if this finding will alter in future prospective RCTs. Socio-Economic Status which is complex and influenced by multiple factors, including education, occupation, income, and social support, was also absent in sub-group outcome analyses although almost 30% of the 247 RCTs measured and reported this as a sample characteristic. Data minimisation, that is collecting necessary data only from participants, is generally expected as part of research ethical approval processes ^52^, yet our findings show that despite collecting these data in 30% of the dataset, none used these data to produce new knowledge, raising ethical concerns of contravening the principle of data minimisation. Furthermore, collecting but not fully utilising diversity data perpetuates the notion that EDI practices are existing as ‘tick box’ activity rather than serving a meaningful function in RCTs. Additionally, collecting and reporting EDI characteristic data as sample characteristic or inclusion/exclusion data is far less tangible and informative for healthcare planning and provision when other analyses for variance are not carried out. Consideration also needs to be given to analyses that account for participants who carry more than one EDI characteristic. 

Living in rural versus urban settings also impacts maternal health. A US nation-wide analysis, for example, found that the maternal mortality rate in rural settings was almost double that of urban locations (Relative Risk 1.93; 95% CI 1.71 to 2.17) ^53^. Addressing health disparities is crucial for improving maternal and societal health and ensuring equitable, tailored access to resources. The lack of consideration for measuring health outcomes and reporting these in sub-group analyses based on EDI characteristics points to a lack of consideration for potential differential treatment effectiveness. Consequently, this prohibits a comprehensive and appropriate translation of RCT evidence across jurisdictions and context-specific maternity healthcare settings and hinders adequate health policy and practice developments. It further leads to gaps in understanding how different populations respond to treatments and may render clinicians who might hope to adopt or utilise RCT findings challenged in being confidently enabled to do so. These findings may also reflect broader issues of inequality in research participation, further perpetuating health disparities because of inadequate explorations and ongoing unknowns.

The EDI characteristic of Age featured prominently in this scoping review. Problematic, however, is the variation in how this was measured and reported, primarily in sample characteristics. This applies to other EDI characteristics also, including Language, Socio-Economic status, Family/Relationship status, Race/Ethnicity, and Education. At an individual RCT level, this might not appear overly problematic, however the challenge arises when trying to synthesise the evidence from RCTs in a systematic review. Methodology for standardising outcome measures in effectiveness trials is well established through the Core Outcome Measures in Effectiveness Trials (COMET) initiative ^54^. A core outcome set represents a minimum set of outcomes that should be measured and reported in all RCTs on a specific health condition. Although many RCTs compare population characteristics at baseline, little attention has been given thus far to standardising a minimum set of EDI characteristics for use in RCTs, including how to measure and report these. Future research is required to address this gap.

Recent decades have witnessed a trend towards increasing maternal age in pregnancy and childbirth. For example, the birth rate among women aged 35–39 has increased in the past 40 years by 272% and for women aged 40–44 years by 318% ^55^. This growing trend impacts individuals and health systems, especially as advanced maternal age, defined historically as >35 years, can lead to increased risks for complications, including hypertension-related conditions, gestational diabetes, preterm birth, fetal growth restriction, and stillbirth ^56^. In this scoping review, few trials explicitly included women >35 years of age although the upper age limit in some trials was not defined ( Table 5). Most trials that reported Age as an inclusion/exclusion criterion used 35 years or below as the upper-level cut-off, with no rationale provided for doing so. This trends against the obligation of equitable recruitment in trials and fails to take account of changing patterns in maternal age in pregnancy and the possible implications of this when evaluating intrapartum treatments or interventions. 

The NIHR component of inclusivity, as applied in this scoping review, necessitates trialists giving due consideration to environments that are supportive towards participation by all who are eligible. We found 15 RCTs that excluded participants on the grounds of Disability. In seven of these RCTs it may appear reasonable to exclude participants based on specific disabilities, for example, the intervention being evaluated engaged virtual reality, showing images or playing music to women during labour, thus excluding participants with visual and hearing impairments. In two RCTs, however, women with hearing or visual impairment were excluded with no apparent indication for this; that is the interventions centred on warm compresses to the perineum during childbirth ^57^ and acupressure ^58^. In a third RCT which evaluated umbilical cord milking, the inclusion criteria necessitated women to be able to communicate verbally, a necessity not explicitly related to the intervention under evaluation ^59^. Similarly, mental health or psychiatric conditions appeared as exclusion criteria in three RCTs, with no clear rationale presented for this ^60– 62^. Research has shown that for women with physical disabilities access to, and experiences of, maternity care is suboptimal ^63^. Trialists, as with all maternity care providers, need to have disability knowledge and awareness and make available, where possible, support services to ensure accessibility and inclusivity for all women in all trials. Language and Education levels also featured as inclusion/exclusion criteria is some trials, although, positively, the numbers of RCTs that excluded participants based on these EDI characteristics were comparatively few (4% and 2% respectively). Nonetheless, in the absence of legitimate reasons, exclusions based on Language and Education contravene inclusive practices, especially when evidence for alternative methods to aid inclusivity (e.g., use of metaphors, plain language, face-to-face discussions with researchers, etc.) are available ^64, 65^. Lastly, the considerable proportion of RCTs (n=90, 36%) limiting inclusion to participants who were pregnant or birthing for the first time without clinical rationale is noteworthy in the context of EDI. Health in pregnancy is influenced by many factors, including that of parity ^66^. Restricting RCT participation solely to nulliparous women may thus not accurately reflect the effect of treatments in all birthing women. Not only does this limit the applicability of the findings, but such restrictions could lead to biases in RCT outcomes as the results will not account for physiological and psychological differences between nulliparous and parous women ^66^. This may further impact and perpetuate disparities in maternal health as important insights that could improve treatments and health outcomes for all women could be missed.

### Strengths and limitations

The strengths of this review include the extensive dataset of 247 RCTs that informed the exploration of 12 pre-specified EDI characteristics and by including intrapartum RCTs only, which enhanced clinical homogeneity and provided scope to successfully undertake the review within a reasonable timeframe was achieved. We acknowledge that including English language published RCTs sourced from electronic database searching only may have reduced the expanse of EDI characteristic exploration; nonetheless, as the purpose of the review was preliminary exploration with the aim of establishing a baseline for discussion and future developments, having a broader dataset based on grey literature and unpublished RCTs would unlikely change or add to the quality or depth of information revealed in the review. We also acknowledge, although discovering a high level of Gender reporting across the included RCTs, this may be related to our assumption of Gender reporting when participants were described as women when, in reality, this could be related to either or both sex and gender. Lastly, we acknowledge that traditional practice in reviews of this type involve independent data extraction of each included record by at least two reviewers independently. We overcame this limitation by undertaking a data extraction pilot process with congruency across extractors achieving 100% on 20 records, and by one reviewer assessing the entire dataset for consistency and standardisation prior to data charting and presentation.

## Conclusion

This scoping review highlights deficits in intrapartum RCTs with respect to measuring and reporting EDI characteristics. There is a critical need for improvements and adaptation of EDI guidelines and toolkits, as well as cultural competence practices in designing, conducting, and reporting intrapartum RCTs. While specific to the healthcare population receiving intrapartum care, the review’s findings are likely to resonate and have applicability to RCTs in other discrete populations. Integrating EDI in RCTs not only meets the obligation of ethical research conduct, but it also serves to enhance the quality and applicability of the findings derived from the RCTs. By adopting more extensive EDI practices, including standardisation of what and how EDI characteristics should be measured and reported, a greater understanding of healthcare treatments, practices, and innovations leading to better benefit and enhanced maternal health equity for all could be achieved.

## Ethics and consent

Ethical approval and consent were not required.

## Data Availability

No data are associated with this article. Repository name: Open Science Framework (OSF), DOI: 10.17605/OSF.IO/7QMD2 (citation ^67^) The project contains the following underlying data: Extended File 1: PRISMA-ScR – complete checklist of items in reporting scoping reviews Extended File 2: Search Terms – complete list of search terms used in all databases Extended File 3: DEF (Data Extraction Form) – Excel File of all data extracted from the included records Extended File 4: Tables S4.1-S4.6 – document of supplementary tables referred to in the manuscript Supplementary tables: Table S4.1: Descriptions of Trial Designs Table S4.2: Maternal Age category descriptors Table S4.3: Education Leve (sample characteristics) Table S4.4: Relationship status (sample characteristics) Table S4.5: Socio-Economic status (sample characteristics) Table S4.6: Rural/Urban Population (sample characteristics) PRISMA Flow Diagram - flow chart of search and selection results License: CC-By Attribution 4.0 International The review is reported as per the Preferred Reporting Items for Reviews Systematic Reviews and Meta-Analyses extension for Scoping (PRISMA-ScR) reporting guideline (Extended File 1: 10.17605/OSF.IO/7QMD2 (citation ^67^) License: CC-By Attribution 4.0 International
