# Supplementary material for: Equality, Diversity and Inclusion characteristics measured or reported in randomised trials of intrapartum interventions: A Scoping Review
Source: HRB Open Res. 2025 Mar 7;7:78. Originally published 2024 Dec 24. [Version 2] doi: 10.12688/hrbopenres.14012.2 (PMC11782935; doi:10.12688/hrbopenres.14012.2)
Supplement: Peer review history

# Reviewer report: Reviewer response for version 1

**DOI:** 10.21956/hrbopenres.15378.r44825

**Published:** 2025-01-30

**Authors:** Lisa Gold

**Affiliations:** 1 Deakin University, Burwood, Victoria, Australia

This scoping review tells us that – in a sample of 247 intrapartum RCTs reported in the last 5 years – most (?all?) reported some EDI data but there are large gaps in EDI reporting. The most commonly-reported was age (222 RCTs) – really a classic sample characteristic that also serves as an EDI characteristic – and the least commonly reported were sexual orientation (0 RCTs) and religion (3 RCTs).

**Why are we interested in EDI?**

 The general point is that we need to know the characteristics of trial participants in order to interpret results. This requires EDI data to be reported in sample characteristics.

 The type of EDI data we are interested in will vary between countries, with the 12 EDI characteristics (Table 2) a logically-selected and comprehensive list that could easily be added to journal/organisational requirements for RCT reporting.

 A more specific point is that RCT evidence should come from population-representative samples, so a second reason to assess EDI data is to highlight inclusion issues in research. As we already know that pregnant women are a highly-excluded group in research, it is a very good choice in this study to assess EDI in the context where this group is by definition included (ie, intrapartum care).

 This scoping review addresses both points. A scoping review is appropriate at this early stage of research in this area. This scoping review is an excellent example of the approach, including pre-published protocol (OSF) and following established methods of conduct and reporting (JBI/PRISMA-ScR). The restriction to more recent evidence is entirely appropriate given the timing of increased awareness/focus on EDI and the review demonstrates a model approach to language of publication where research resources are limited (ie, no language restriction in search but exclude from review and discuss impact of this).

**What do we learn from this review?**

 The results of the scoping review demonstrate variation across EDI characteristics in the extent of reporting, and variation of categorizations within EDI characteristics. Table 4 makes a good point that some EDI (disability) is reported only in the context of inclusion/exclusion criteria.

 The discussion highlights important considerations for EDI reporting. The section on (non)use of EDI data in analysis should include cautions around the presentation of sub-group analysis that may be misinterpreted in the context of low statistical power – at least that this may well be the reason that EDI data is reported in sample characteristics but not used in (sub-group) analysis. This would link nicely into the issue of variance of categorization, as EDI-based sub-group analysis could be addressed more appropriately in meta-analyses across RCTs, but that requires consistent EDI categorization, which is difficult when categorization is country-specific.

**Minor comments for presentation:**

 Results: While the benefit of open-source publishing is that you can include more detail in a paper, there is quite a lot of detail presented on the RCT design that is not relevant to the EDI topic and could be edited (Characteristics of included trials) or moved to supplementary material (Figure 3). Similarly, there could be less detail presented on the variation of reporting within gender (last paragraph p7) and Table 3 is enough to illustrate this variation in the main paper, with Tables 5-6 and Figures 5-6 moved to supplementary material?

 Objectives 1 & 2: please add to first sentence the number (of 247 RCTs) that reported at least one EDI characteristic. Potentially you could also add a graphic of the distribution of the 247 RCTs across the number of EDI characteristics reported, to give a visual representation of the depth of EDI reporting?

 Figure 4: Please add numbers to Figure 4 – it is a main result to show the number of trials reporting each of the 12 EDI characteristics. Also if possible keep it in the same order as Table 2 (and add sexual orientation back in, as n=0).

**Typos:**

 P5 Quality Appraisal: extent not extend

 P8 There is one “RCTS” that should be have a small “S”

Are the rationale for, and objectives of, the Systematic Review clearly stated?

Yes

Is the statistical analysis and its interpretation appropriate?

Not applicable

Are sufficient details of the methods and analysis provided to allow replication by others?

Yes

Are the conclusions drawn adequately supported by the results presented in the review?

Yes

Reviewer Expertise:

Public Health, Health Economics

I confirm that I have read this submission and believe that I have an appropriate level of expertise to confirm that it is of an acceptable scientific standard.

# Response

**Published:** 2025-02-28

**Authors:** Valerie Smith

**Affiliations:** Nursing, Midwifery, Health Sys, University College Dublin, Dublin, Leinster, Ireland

Thank you very much for taking the time to review our work and for your thoughtful comments. These are much appreciated. We have edited the original version to address your comments as follows:

1. We are grateful for the point you made about how the (non)use of EDI data in analysis should include cautions around the presentation of sub-group analysis that may be misinterpreted in the context of low statistical power in the Discussion section is valued. We have addressed this with the addition of the following text (Discussion section, end of paragraph 3):

**Response: **Countering this, we acknowledge that reporting of EDI data in sample characteristics but not in sub-group analyses could relate to maintaining statistical power in RCTs, yet EDI-based analyses could be addressed in meta-analyses across RCTs.  For this to occur, however, consistent EDI categorization is required; a variation challenge identified in this scoping review.

1. Results: While the benefit of open-source publishing is that you can include more detail in a paper, there is quite a lot of detail presented on the RCT design that is not relevant to the EDI topic and could be edited (Characteristics of included trials) or moved to supplementary material (Figure 3).

**Response:** We have now reduced the details in the section on Characteristics of included trials including some of the text on country of origin, trial design, RCT start dates and duration periods, and the numbers included in the RCTs. Figure 3 has been removed from the manuscript and is now included in Extended File 4 (Figure S4.1).  

1. Similarly, there could be less detail presented on the variation of reporting within gender (last paragraph p7) and Table 3 is enough to illustrate this variation in the main paper, with Tables 5-6 and Figures 5-6 moved to supplementary material?

**Response**: We have removed the text related to the gender of the infant from the main manuscript, although we have retained illustrative Figures 5-6 (now Figures 4 and 5) in the main paper.

1. Objectives 1 & 2: please add to first sentence the number (of 247 RCTs) that reported at least one EDI characteristic. Potentially you could also add a graphic of the distribution of the 247 RCTs across the number of EDI characteristics reported, to give a visual representation of the depth of EDI reporting?

**Response:** We have added the following sentence to the start of this section: Of the 247 RCTs, five did not report any of the 12 EDI characteristics. We refrained though from creating a new graphic, as we feel Figure 2, Table 4 and Extended File X collectively help capture the depth of EDI reporting.

1. Figure 4: Please add numbers to Figure 4 – it is a main result to show the number of trials reporting each of the 12 EDI characteristics. Also, if possible, keep it in the same order as Table 2 (and add sexual orientation back in, as n=0).

**Response:** We have now added numbers into Figure 4 (now Figure 3) and added in Sexual Orientation. The list is in the same order as Table 2, only from the bottom up (Excel appears to default to this for this type of bar chart)

1. Typo P5 Quality Appraisal: extent not extend

Response: Thank you, type corrected

1. Typo P8 There is one “RCTS” that should have a small “S”

Response: Thank you, type corrected

# Reviewer report: Reviewer response for version 1

**DOI:** 10.21956/hrbopenres.15378.r44558

**Published:** 2025-01-10

**Authors:** Jennifer Vanderlaan

**Affiliations:** 1 University of Nevada Las Vegas, Las Vegas, USA

Thank you for the opportunity to read and review this manuscript. The manuscript presents a scoping review of randomized controlled trials of intrapartum interventions to determine the extent to which diversity, equity, and inclusion are incorporated into the studies. I agree this is an important question, and the results highlight opportunities for researchers to improve the study of intrapartum interventions. As this is a scoping review, the authors do not present statistics. The study is presented according to the PRISMA guidelines. Overall I feel this article is unique and contributes to the general knowledge and recommend its publication. Below I query a few minor issues that could be clarified by the authors to assist the readers.

1. The authors indicated that an automated filter was used to remove duplicates and ineligible during the initial article collection. Was AI also used during the Title and abstract screening?
2. Some of the figures lack the information needed to interpret the figure. The caption for figure 2 does not include the total n. Figures 5 and 6 present only percentages without the count or total n to help the reader interpret the result.  

Are the rationale for, and objectives of, the Systematic Review clearly stated?

Yes

Is the statistical analysis and its interpretation appropriate?

Not applicable

Are sufficient details of the methods and analysis provided to allow replication by others?

Yes

Are the conclusions drawn adequately supported by the results presented in the review?

Yes

Reviewer Expertise:

Midwifery, Maternal Health Services

I confirm that I have read this submission and believe that I have an appropriate level of expertise to confirm that it is of an acceptable scientific standard.

# Response

**Published:** 2025-02-28

**Authors:** Valerie Smith

**Affiliations:** Nursing, Midwifery, Health Sys, University College Dublin, Dublin, Leinster, Ireland

Thank you very much for taking the time to review our work and for your thoughtful comments which are much appreciated. We have edited the original version to address your comments as follows:

1. The authors indicated that an automated filter was used to remove duplicates and ineligible during the initial article collection. Was AI also used during the Title and abstract screening?

Response: AI was used only for capturing duplicates and not for screening; that is title and abstract screening, and full text screening were undertaken by the reviewer team.  We have clarified this by altering the sentence to read: Title and abstract screening of 17,176 records was thus undertaken by a member of the review team and 16,452 records were excluded as ineligible.

1. Some of the figures lack the information needed to interpret the figure. The caption for figure 2 does not include the total n. Figures 5 and 6 present only percentages without the count or total n to help the reader interpret the result.  

For Figure 2, we have added (n=240) to the caption, based on the RCTs where country of origin was known. For Figures 5 and 6 (now Figures 4 and 5 based on comments from the second reviewer), we have similarly added the total n RCTs in the Figure captions and added the counts that each % relates to in the Figures.
